# Effects of releasing ankle joint during electrically evoked cycling in persons with motor complete spinal cord injury

**DOI:** 10.1038/s41598-024-56955-w

**Published:** 2024-03-18

**Authors:** Puteri Nur Farhana Hamdan, Nur Azah Hamzaid, Nazirah Hasnan, Nasrul Anuar Abd Razak, Rizal Razman, Juliana Usman

**Affiliations:** 1 Department of Biomedical Engineering, Faculty of Engineering, Universiti Malaya, 50603 Kuala Lumpur, Malaysia; 2 Department of Rehabilitation Medicine, Faculty of Medicine, Universiti Malaya, 50603 Kuala Lumpur, Malaysia; 3 Centre for Sport & Exercise Sciences, Universiti Malaya, 50603 Kuala Lumpur, Malaysia; 4 Department of Biomedical Engineering, Faculty of Engineering, Centre of Applied Biomechanics, Universiti Malaya, 50603 Kuala Lumpur, Malaysia

**Keywords:** Functional electrical stimulation, Paraplegics, Pedal power, Ankle biomechanics, Rehabilitation, Anatomy, Engineering

## Abstract

Literature has shown that simulated power production during conventional functional electrical stimulation (FES) cycling was improved by 14% by releasing the ankle joint from a fixed ankle setup and with the stimulation of the tibialis anterior and triceps surae. This study aims to investigate the effect of releasing the ankle joint on the pedal power production during FES cycling in persons with spinal cord injury (SCI). Seven persons with motor complete SCI participated in this study. All participants performed 1 min of fixed-ankle and 1 min of free-ankle FES cycling with two stimulation modes. In mode 1 participants performed FES-evoked cycling with the stimulation of quadriceps and hamstring muscles only (QH stimulation), while Mode 2 had stimulation of quadriceps, hamstring, tibialis anterior, and triceps surae muscles (QHT stimulation). The order of each trial was randomized in each participant. Free-ankle FES cycling offered greater ankle plantar- and dorsiflexion movement at specific slices of 20° crank angle intervals compared to fixed-ankle. There were significant differences in the mean and peak normalized pedal power outputs (POs) [F(1,500) = 14.03, p < 0.01 and F(1,500) = 7.111, p = 0.008, respectively] between fixed- and free-ankle QH stimulation, and fixed- and free-ankle QHT stimulation. Fixed-ankle QHT stimulation elevated the peak normalized pedal PO by 14.5% more than free-ankle QH stimulation. Releasing the ankle joint while providing no stimulation to the triceps surae and tibialis anterior reduces power output. The findings of this study suggest that QHT stimulation is necessary during free-ankle FES cycling to maintain power production as fixed-ankle.

## Introduction

Following muscle atrophy, muscle size and joint range of movement (ROM) decrease significantly<Superscript><CitationRef ^1^. This is especially true following prolonged immobilization due to spinal cord injury (SCI)^2^. Functional electrical stimulation (FES)-evoked cycling has reported improved muscle strength^3^, muscle size^4^, and joint ROM^5^. FES-evoked cycling has also gained much attention due to its safety and practicality^6^.

FES is applied to the peripheral nerve^7^ to artificially activate paralyzed muscles^8^ due to spinal cord injury. The goal of FES-evoked cycling is to produce the highest possible power to maximize the merits of health benefits^9^. However, the mechanical power and mechanical efficiency are very low^10^ compared to voluntary cycling^11^. Due to unfavorable biomechanics, weakened or paralyzed muscles, and the fact that FES can partially activate only superficial muscles when using surface electrodes, the mechanical power produced by a person with SCI is typically ten times lower than that of an average healthy cyclist^12^. The synchronous stimulation of motor units that are typically employed during FES-evoked cycling using surface electrodes leads to imprecise flexor and extensor coordination and results in less efficient cycling biomechanics^13^ and earlier muscle fatigue^7^. Biomechanical inefficiency becomes the most prominent factor in reducing mechanical power^14^.

In the standard setup for FES-evoked cycling, the muscles activated are the quadriceps, hamstring, and gluteus^15^, while the ankle joints are immobilized using solid ankle–foot orthoses (AFO)^16,17^. FES applied to the overlying key muscles in continuous sequence depending on the pedal angle would generate pedaling power that could influence the health and fitness of the users^18^. The primary power source is the knee extensors i.e., the quadriceps, followed by the hamstrings as knee flexors^13^. However, the power generated from the knee extensors of the quadriceps and knee flexors of the hamstring were approximately equal in a minority of persons with SCI.

Nevertheless, ankle joint biomechanics in FES-evoked cycling have received little attention^19^. Ankle positioning during cycling is one of the more important factors for effective pedaling^20,21^, as the overall lower limb biomechanics are affected by the ankle patterns^22^. Typically, persons with SCI have weak ankle muscles^23^ or no muscle power and do not have the ability to control ankle muscle contractions^24^ and movement. During FES-evoked cycling, solid AFOs are often used to limit and control the ankle motion^25^ at 90° angle^26^ and provide shank stability^19^ that restricts leg movements in the sagittal plane^27^. Ferrante et al.^28^ reported that the calf muscles generate limited knee flexion action due to the presence of solid AFO, which might reduce the maximum power during FES-evoked cycling in these individuals. The stimulation of lower leg muscles while fixing the ankle joint during FES-evoked cycling in individuals with SCI produced a non-significant difference in the mechanical work compared to the stimulation of upper leg muscles alone^29^. This is because the gastrocnemius muscle was the only lower leg muscle that had the potential to generate mechanical work during FES-evoked cycling while fixing the ankle joint. A simulation used to determine the electrical stimulation timing patterns including the lower leg muscles, indicated that the gastrocnemius activity did not result in a net gain in mechanical work to drive the crank^30^. Hakansson et al. reported a similar finding in able-bodied cyclists despite their ability to flex and extend the ankle joint^31^. Theoretically, the power produced during FES-evoked cycling can be improved by up to 14% by releasing the ankle joint and adding the stimulation of shank muscles (triceps surae and tibialis anterior), only with the tuning of the contact point between the foot and pedal to the relative strength of the ankle plantar flexors of the triceps surae compared to the fixed-ankle joint^32^. Fornusek et al.^33^ reported that freeing the ankle joint during FES-evoked cycling was found to be safe. The combination of shank muscle stimulation and freeing the ankle joint movement may improve ankle flexibility^33^. However, to date, no studies have experimentally investigated the effect of releasing the ankle joint on power production during FES-evoked cycling in persons with SCI. Therefore, the purpose of this study is to investigate the effect of releasing the ankle joint on pedal power production during FES-evoked cycling in persons with SCI. We hypothesized that freeing the ankle joint and adding stimulation of shank muscles in individuals with SCI would elevate the pedal PO by at least 10% compared to fixed-ankle FES-evoked cycling.

## Methods

A quasi-experimental research design was adopted whereby participants performed all trials in different conditions (fixed- and free-ankle, with different muscle stimulation), but their order of trials was randomized.

### Participants

Seven persons with complete SCI (ASIA impairment scale (AIS) A and B (i.e., motor complete paralysis), lesion level between C5 to T11) participated in the study (Table 1). Based on pilot trials, the consistency of biomechanical performance in all tested conditions indicated that the statistical power is sufficient with 7 subjects^34^, given the highly predictable output due to the mechanical constraints on the legs. Participants were invited as volunteers and were screened according to the AIS assessment by clinicians to meet the inclusion criteria. All participants provided their written informed consent before participating in the study. Participants with no previous or ongoing record of neuromuscular, musculoskeletal, rheumatological, cardiovascular disorder, or orthopedic lower limb injuries were included. Prior to the experiment, all the participants were trained with FES-evoked cycling for at least 12 weeks^35^. The participants were trained in two sessions per week. To ensure that all the upper and lower leg muscles were equally trained with FES without limiting the ankle joint movement, each training session required the participants to cycle in a free-ankle setup with the stimulation of quadriceps, hamstring, tibialis anterior, and triceps surae muscles; referred to as QHT stimulation at their maximum stimulation intensity for at least 30 min. This study was approved by the local Medical Ethics Committee, University of Malaya Medical Centre, University Malaya, Kuala Lumpur, Malaysia (Ref No.: 1003.14(1); 22/07/2013). All methods were performed in accordance with the Declaration of Helsinki.

**Table 1:** Physical characteristics of the SCI participants.

Participant	Age (years)	Gender	Height (m)	Weight (kg)	Level of injury	AIS	Time since injury (years)	Maximum stimulation intensity (mA)
1	49	F	1.62	82.0	T4	B	26	100
2	51	M	1.74	79.6	T1	A	13	100
3	30	M	1.71	62.4	C7	B	16	100
4	36	M	1.70	75.9	C6	A	19	100
5	59	M	1.73	80.0	C5–C7	B	6	60
6	46	M	1.79	71.6	C6–C7	B	5	100
7	61	M	1.72	60.5	T10–T11	A	15	60
Mean ± standard deviation (SD)	47.4 ± 11.3		1.7 ± 0.1	73.1 ± 8.7			14.3 ± 7.3	86.7 ± 20.7

### Instrumentation

**Figure 1:**
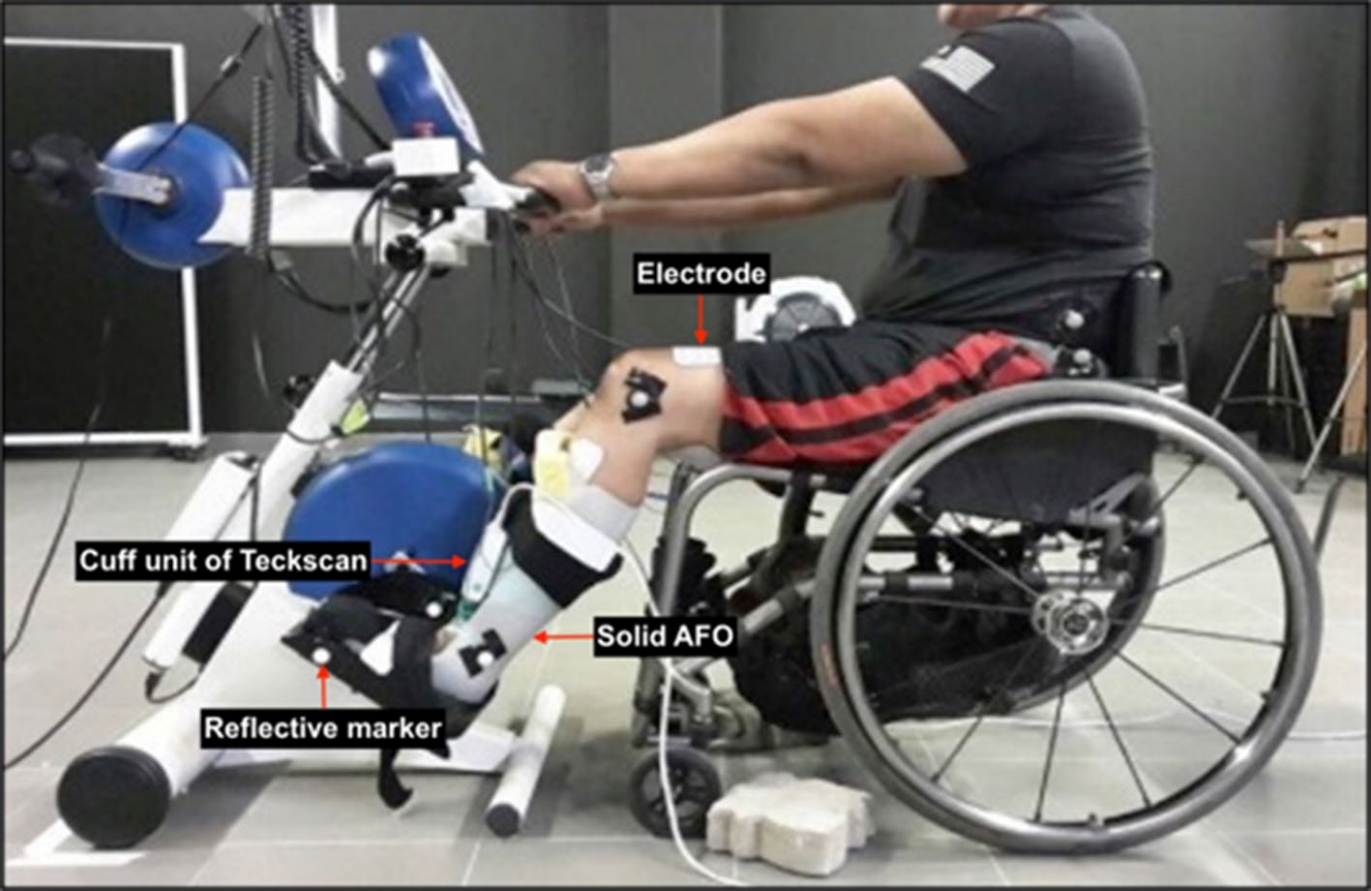
Set up for fixed-ankle FES-evoked cycling. Shown is the placement of markers over the fifth metatarsophalangeal and ankle joints on the solid AFO. Electrodes were placed on the quadriceps, hamstrings, tibialis anterior, and triceps surae muscles.

A MOTOmed Viva 2 FES cycle ergometer (RECK-Technik GmbH, Betzenweiler, Germany) was utilized in this study (Fig. 1). Self-adhesive gel-backed surface stimulating electrodes were placed over the belly of quadriceps, hamstring, tibialis anterior, and triceps surae muscles that were stimulated. For quadriceps, the proximal electrode was placed 1/3 of the distance from the inguinal line to the superior patellar border and the distal electrode was placed 6–8 cm proximally to the patellar border^13^. For the hamstrings, the proximal electrode was placed 2–4 cm below the gluteal crease and the distal electrode was placed 4–5 cm above the popliteal space^13^. For tibialis anterior, the proximal electrode was placed 2 cm below the fibula head and the distal electrode was placed 4–5 cm from the ankle joint. For triceps surae, the proximal electrode was placed 4–5 cm below the popliteal space and the distal electrode was placed 4–5 cm from the ankle joint. Stimulating electrode placement was kept consistent between trials. To keep the placement of the stimulating electrodes consistent between trials, only one similar person applied the stimulating electrodes on the participants during training and experimental sessions. In addition, the measurement of the stimulating electrode placement was recorded for each participant. An in-shoe F-scan system (Teckscan Incorporated, Boston, Massachusetts) was placed under the participants’ feet and connected to a cuff unit that linked the foot sensors to a computer via a 10 m cable^36^. A pair of solid AFOs was used to restrict the ankle joint movement at a neutral position (90°). The lower legs of each participant were placed in the solid AFO that was fixed to the pedal during fixed-ankle FES-evoked cycling. No AFO was used during free-ankle FES-evoked cycling to allow the ankle to move from a neutral position to dorsi-plantarflexion. The pedal spindle was attached to the top middle part of the foot. The seat position from the crank axle was adjusted and recorded for each participant so that the knee extension did not exceed 150–160° at the bottom dead center^13^. The knee extension angle was measured using an analogue goniometer. The hip, knee, ankle, pedal, and crank kinematics were recorded using 3D motion analysis systems (Qualisys AB, Gothenburg, Sweden, and Vicon, Oxford, UK). During fixed-ankle FES-evoked cycling, the marker placement for the ankle joint was on the AFO.

### Leg muscles stimulation pattern

**Figure 2:**
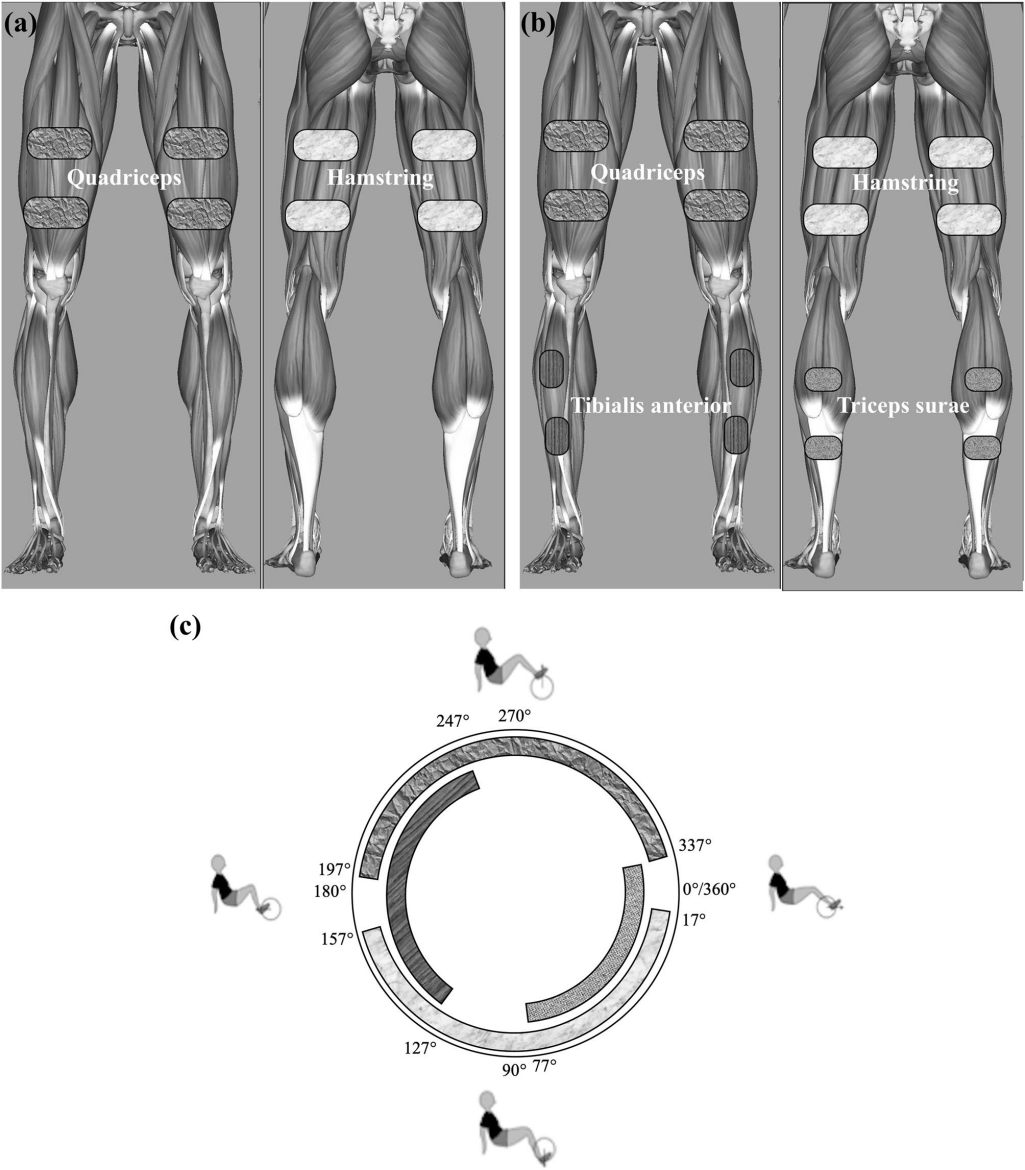
Two stimulation modes of FES-evoked cycling were used in this study; (**a**) QH stimulation; (**b**) QHT stimulation; and (**c**) stimulation angle. Image adapted from the software 3D Anatomy Learning (Version 3.9, Education Mobile) (open-source project).

Two sets of stimulation modes were determined for comparison. In mode 1, the participants performed FES-evoked cycling with the stimulation of quadriceps and hamstring muscles; i.e. QH stimulation (Fig. 2a). In mode 2, the participants performed FES-evoked cycling with QHT stimulation (Fig. 2b).

The stimulation angle of each muscle was fixed between the participants and within the cycling modes based on an earlier study^19^ (Fig. 2c). The lower leg muscles’ stimulation timing i.e., of the tibialis anterior, and triceps surae, was set to encourage plantar- and dorsiflexion of the ankles (quadriceps: 197° to 337°, hamstring: 17° to 157°, tibialis anterior: 127° to 247°, and triceps surae: 337° to 77°). The gluteal muscles were not stimulated at all in this study as it was reported to produce no measurable crank torques in most subjects^13^, and also due to the limited number of stimulation channels available on the FES cycling device.

#### Experimental protocol

Each participant completed all 2 sets of trials in randomized order. Trial set 1 required the participants to perform fixed-ankle FES-evoked cycling, while trial set 2 required the participants to perform free-ankle FES-evoked cycling. Each trial set required the participants to perform FES-evoked cycling with 2 different stimulation modes, i.e., mode 1 and mode 2. The order of each trial set; fixed-ankle QH stimulation, free-ankle QH stimulation, fixed-ankle QHT stimulation, and free-ankle QHT stimulation, was randomized for each participant. For each trial set and mode, the participants performed 1 min of passive cycling (warm-up), 1 min of FES-evoked cycling^37^, 1 min of passive cycling (cool down), and 10 min of resting phase. Steady-state was identified when the participants reached constant cadence. The participants performed 2 sets of trials in two sessions. Each session was separated by at least 48 h of recovery period to prevent excessive muscle fatigue effect^38^. The participants performed FES-evoked cycling at 50 revolutions per minute (rpm). Fixed stimulation pulse width (300 µs) and frequency (30 Hz), and the highest tolerance stimulation intensity (up to 120 mA) were applied by an 8-channel stimulator (RehaStim ScienceMode, HASOMED GmbH, Germany) during all trial sets. Prior to the experiment, the highest tolerance stimulation intensity was recorded for each participant at the end of the training periods (Table 1). The stimulation intensity would be increased gradually from the beginning of the training session. For participants with AIS B, their highest tolerance stimulation intensity was defined when they felt pain or discomfort. For participants with AIS A, their highest tolerance stimulation intensity was defined when there was an initial significant movement induced by the FES.

### Data acquisition and processing

The pedal power output (PO) and the hip, knee, and ankle joints kinematics of each trial set were recorded at 120 Hz, displayed in real-time using software (Tekscan Incorporated, Boston, Massachusetts) and 3D motion analysis systems (Qualisys AB, Gothenburg, Sweden, and Vicon, Oxford, UK) to store data into a PC for offline analysis. These data were synchronously recorded and stored for the entire 1 min cycling period. 10 complete cycles of 0° to 360° crank angle from the last 20 s of the data, where the cycling pace was most consistent, were analyzed^13,39^. The mean and peak pedal POs (W) were normalized (W/W (%)) to the maximum PO of overall performance from 0° to 360° crank angle for each participant. The hip, knee, and ankle angles captured were derived to generate hip, knee, and ankle ROMs. The mean and peak normalized pedal POs, hip, knee, and ankle joints ROMs of each trial set were then averaged for every 20° crank angle for further analyses. The initial crank angle across 20° crank intervals is represented as 20° (the averages of 0° to 20°). The mean and peak normalized pedal POs, hip, knee, and ankle joints’ ROMs for each 20° slice from 0° to 360° crank angle were derived for further analyses.

### Statistical analysis

Two-way repeated measures analysis of variance (ANOVA) was performed to analyze the difference in the ankle movement during FES-evoked cycling within the four conditions, i.e., (a) fixed-ankle QH stimulation, (b) free-ankle QH stimulation, (c) fixed-ankle QHT stimulation, and (d) free-ankle QHT stimulation, in terms of its PO and ROM. The two-way ANOVA analyses of each condition were derived from each of the 20° crank angle positions, as 18 segments of 0° to 360° crank angle. In addition, an LSD post hoc test was conducted to compare all PO and ROM generated by the four conditions of ankle movement during FES-evoked cycling for each 20° slice from 0° to 360° crank angle. All statistical analyses were performed using SPSS software (IBM SPSS Statistics version 20, New York, USA). Statistical significance was determined at an alpha (α) = 0.05 (p < 0.05).

## Results

In overall cycling performance from 0° to 360° crank angle, fixed- and free-ankle FES-evoked cycling produced mean pedal POs that ranged from 1.2 ± 0.5 W to 27.1 ± 16.8 W (minimum PO ± SD to maximum PO ± SD), and from 0.6 ± 0.3 W to 28.4 ± 8.8 W, respectively (Table 2). These values were derived from 2 different muscle stimulation settings i.e., QH and QHT, for each condition. For QH only stimulation, the pedal POs generated during FES-evoked cycling with fixed- and free-ankle ranged between 1.5 ± 0.3 W to 22.4 ± 17.6 W and 0.6 ± 0.3 W to 22.8 ± 16.4 W, respectively. On the other hand, when all QHT were stimulated, the fixed- and free-ankle pedal POs ranged between 1.2 ± 0.5 W to 27.1 ± 16.8 W and from 1.5 ± 0.8 W to 28.4 ± 8.8 W, respectively.

**Table 2:** The range of raw pedal PO obtained between fixed- and free-ankle FES-evoked cycling with QH and QHT stimulation modes, generated from 0° to 360° crank angle.

Participant	Pedal PO (W) [mean ± SD (min–max)]			
	Fixed-ankle QH stimulation	Free-ankle QH stimulation	Fixed-ankle QHT stimulation	Free-ankle QHT stimulation
1	4.8 ± 2.5 (0.3–8.7)	1.5 ± 0.9 (0.2–6.2)	3.1 ± 3.2 (0.1–15.1)	1.5 ± 0.8 (0.1–4.3)
2	22.4 ± 17.6 (1.7–104.9)	22.8 ± 16.4 (0.6–69.2)	27.1 ± 16.8 (3.5–79.8)	28.4 ± 8.8 (9.4–51.7)
3	17.9 ± 14.7 (0.9–113.5)	7.6 ± 10.0 (0.2–68.0)	16.1 ± 7.8 (2.3–38.2)	7.3 ± 6.2 (0.6–43.9)
4	6.1 ± 3.6 (0.6–23.7)	5.1 ± 4.3 (0.03–23.8)	7.0 ± 5.4 (1.5–24.6)	7.2 ± 6.8 (0.2–26.7)
5	3.1 ± 2.7 (0.3–20.1)	2.5 ± 2.8 (0.1–21.6)	4.1 ± 3.4 (0.3–16.1)	3.3 ± 2.1 (0.04–16.1)
6	7.6 ± 4.6 (0.2–21.6)	14.2 ± 6.5 (2.3–39.1)	19.2 ± 6.9 (7.0–40.3)	26.6 ± 9.4 (9.1–54.9)
7	1.5 ± 0.3 (0.6–2.5)	0.6 ± 0.3 (0.04–1.3)	1.2 ± 0.5 (0.4–2.9)	1.7 ± 0.5 (0.8–3.6)

**Figure 3:**
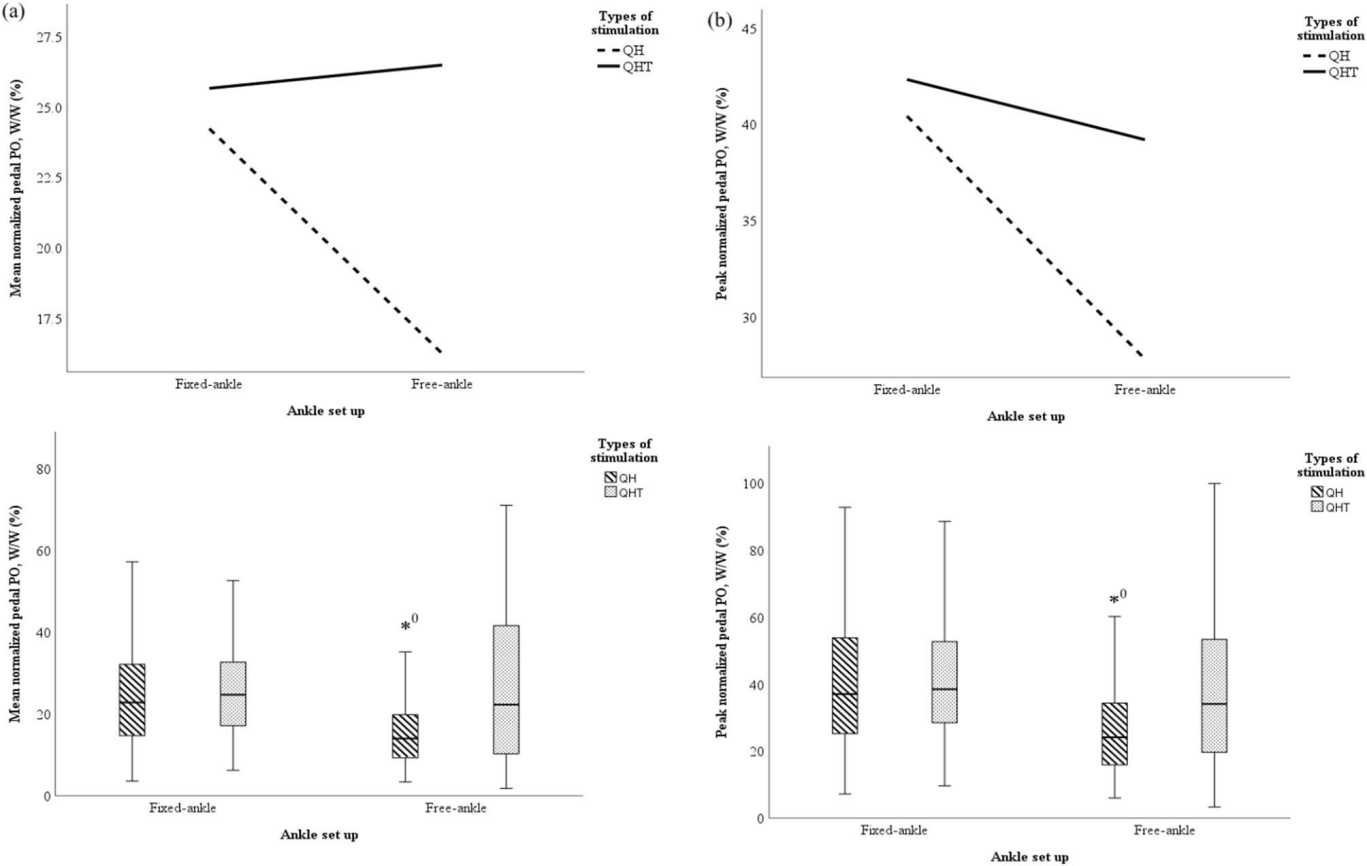

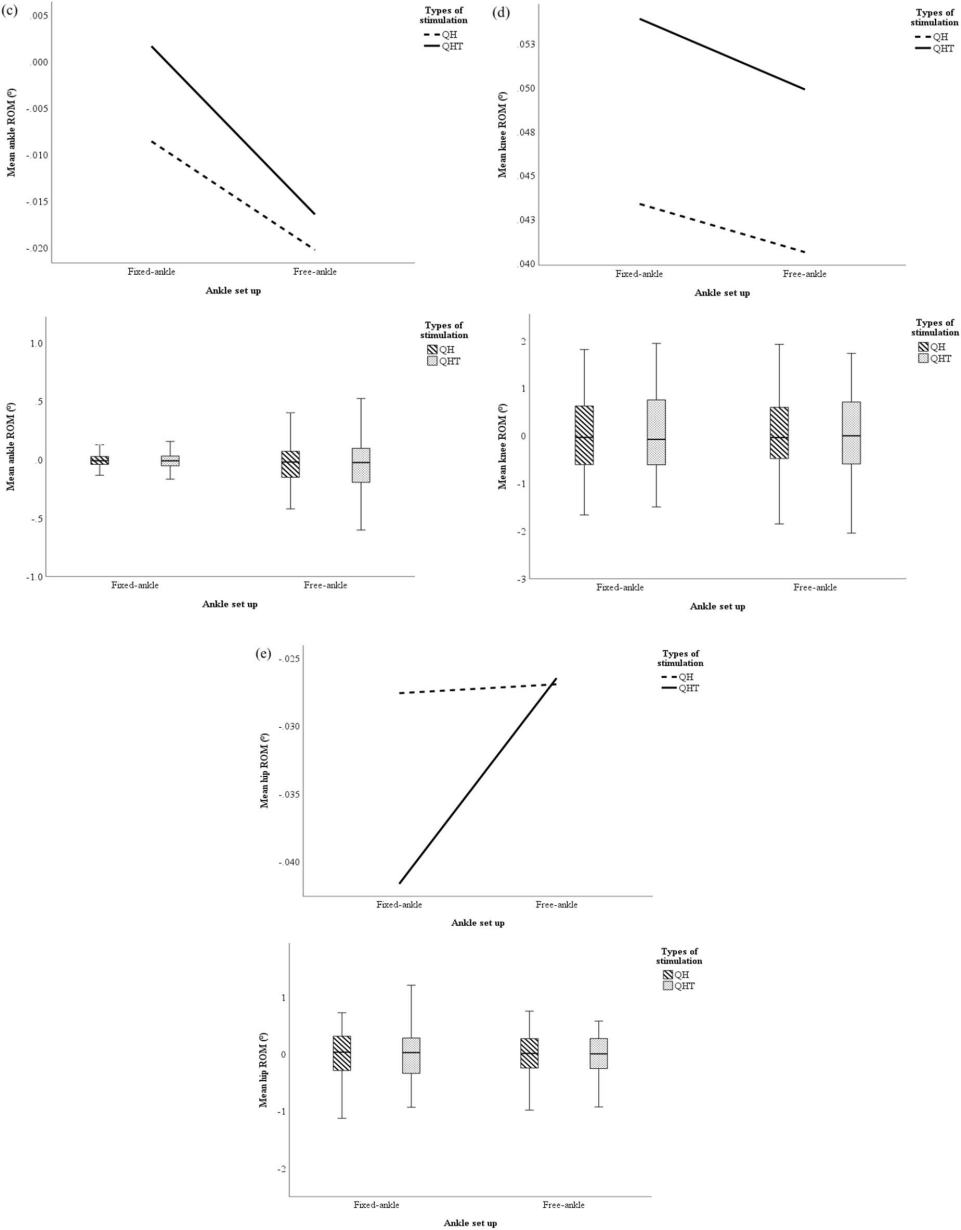
The interaction and boxplot of the effect of fixed- and free-ankle FES-evoked cycling with QH and QHT stimulations across each slice of 20° crank angle intervals on (**a**) mean normalized pedal PO; (**b**) peak normalized pedal PO; (**c**) mean ankle ROM; (**d**) mean knee ROM; and (**e**) mean hip ROM. *^0^Denotes p < 0.05 between free-ankle QH stimulation compared to the other settings.

A two-way ANOVA was performed to analyze the effect of fixed- and free-ankle, and QH and QHT stimulations during FES-evoked cycling on the mean and peak normalized pedal POs across 18 segments of 0° to 360° crank angle (Fig. 3a,b). The present study revealed that there was a statistically significant interaction between the effect of fixed- and free-ankle, and QH and QHT stimulations during FES-evoked cycling on the mean [F(1,500) = 14.03, p < 0.01, ƞ_p_^2^ = 0.027] and peak normalized pedal POs [F(1,500) = 7.111, p = 0.008, ƞ_p_^2^ = 0.014] for each slice of 20° crank angle intervals. Further analysis showed that the interaction between free-ankle, and QH and QHT stimulations significantly altered the mean and peak pedal POs (p < 0.01), but there were no differences between fixed-ankle, and QH (p = 0.389) and QHT stimulations (p = 0.451). The present study also revealed that there were significantly lower mean and peak normalized pedal POs (16.2 ± 9.8% and 27.8 ± 16.8%, respectively) in the free-ankle QH stimulation compared to the rest of the setting (Fig. 3a,b) for each slice of 20° crank angle intervals.

In overall cycling performance across 18 segments of 0° to 360° crank angle, fixed- and free-ankle FES-evoked cycling generated ankle ROM that ranged from − 0.007 ± 0.1° to 0.1 ± 0.6°, and from − 0.001 ± 0.4° to 0.02 ± 0.3°, respectively. The ankle ROM during fixed- and free-ankle QH stimulations ranged from − 0.004 ± 0.02° to 0.002 ± 0.04° and − 0.04 ± 0.3° to 0.03 ± 0.2°, respectively. On the other hand, the ankle ROM generated during fixed- and free-ankle QHT stimulations ranged between − 0.007 ± 0.1° to 0.1 ± 0.6° and − 0.001 ± 0.4° to 0.01 ± 0.1°, respectively. The present study revealed that free-ankle setting allowed not significantly greater ankle variations for both QH (− 0.020 ± 0.3°) and QHT stimulations (− 0.016 ± 0.3°), compared to fixed-ankle QH (− 0.009 ± 0.1°) and QHT stimulations (0.002 ± 0.3°) across each slice of 20° crank angle intervals (Fig. 3c). On the other hand, the knee ROM produced during fixed- and free-ankle FES-evoked cycling were ranged from − 0.01 ± 0.8° to 0.1 ± 0.6° and 0.001 ± 0.1° to 0.1 ± 0.6°, respectively. Whereas the hip ROM obtained during fixed- and free-ankle FES-evoked cycling ranged from − 0.05 ± 0.5° to 0.01 ± 0.3° and − 0.005 ± 0.1° to 0.03 ± 0.2°, respectively.

A two-way ANOVA was also performed to analyze the effect of fixed- and free-ankle, and QH and QHT stimulations during FES-evoked cycling on the mean ankle, knee, and hip ROMs for each slice of 20° crank angle intervals (Fig. 3c–e). The present study revealed that there was no statistically significant interaction between the effect of fixed- and free-ankle, and QH and QHT stimulations during FES-evoked cycling on the ankle [F(1,500) = 0.020, p = 0.888, ƞ_p_^2^ < 0.001], knee [F(1,500) = 0.00, p = 0.993, ƞ_p_^2^ < 0.001], and hip ROMs [F(1,500) = 0.043, p = 0.836, ƞ_p_^2^ < 0.001] for each slice of 20° crank angle intervals. Further analysis showed that the interaction between fixed-ankle and free-ankle, and QH and QHT stimulations did not significantly alter the mean ankle (p = 0.751 and p = 0.905, respectively), knee (p = 0.979 and p = 0.969, respectively), and hip ROMs (p = 0.777 and p = 0.993, respectively).

### Kinetics and kinematics change throughout 360° of crank angle

**Figure 4:**
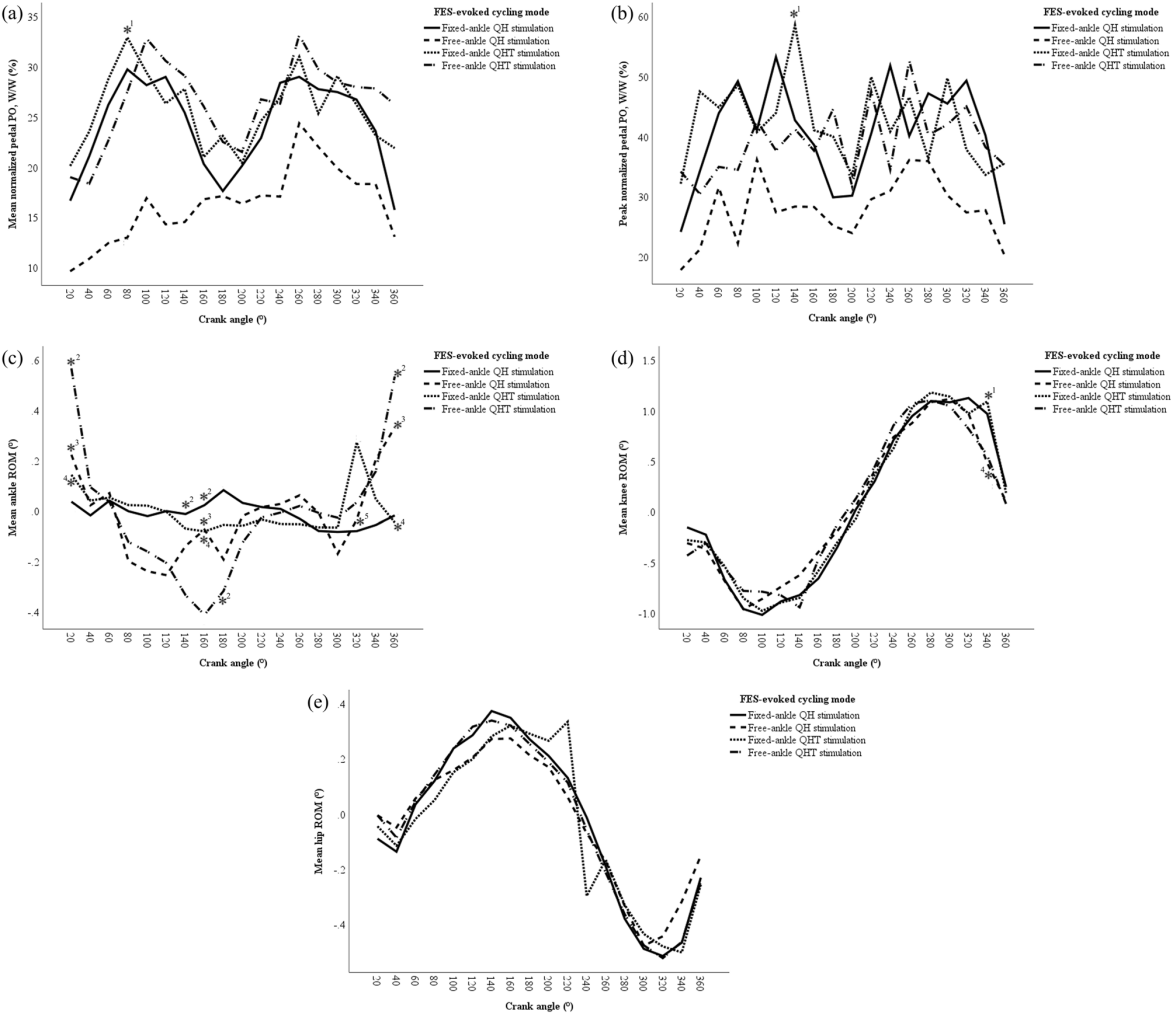
The PO and ROM generated during fixed- and free-ankle FES-evoked cycling with QH and QHT stimulations by each slice of 20° crank angle position from 0° to 360°. (**a**) mean normalized pedal PO; (**b**) peak normalized pedal PO; (**c**) mean ankle ROM; (**d**) mean knee ROM; and (**e**) mean hip ROM. *^1^Denotes p < 0.05 between free-ankle QH stimulation and fixed-ankle QHT stimulation, *^2^denotes p < 0.05 between free-ankle QHT stimulation and fixed-ankle QH stimulation, *^3^denotes p < 0.05 between free-ankle QHT stimulation and free-ankle QH stimulation, *^4^denotes p < 0.05 between free-ankle QHT stimulation and fixed-ankle QHT stimulation, and *^5^denotes p < 0.05 between fixed-ankle QHT stimulation and fixed-ankle QH stimulation.

The mean and peak normalized pedal POs generated during FES-evoked cycling with fixed- and free-ankle QH stimulation, and fixed- and free-ankle QHT stimulation for each slice of 20° crank angle intervals are presented in Fig. 4a,b. Simple main effects analysis showed that all FES-evoked cycling conditions i.e., between fixed- and free-ankle, and QH and QHT stimulations, have a statistically significant effect on mean and peak pedal POs (p < 0.001) for each slice of 20° crank angle intervals. The present study revealed that there was a significant difference in the mean normalized pedal PO generated between free-ankle QH stimulation and fixed-ankle QHT stimulation (p = 0.037) at the crank angle of 80° (Fig. 4a).

There was also a significant difference in the peak normalized pedal PO generated between free-ankle QH stimulation and fixed-ankle QHT stimulation (p = 0.033) at the crank angle of 140° (Fig. 4b).

The mean ankle, knee, and hip ROMs generated during FES-evoked cycling with fixed- and free-ankle QH stimulation, and fixed- and free-ankle QHT stimulation for each slice of 20° crank angle intervals are presented in Fig. 4c–e. Simple main effects analysis revealed that there were significant differences in the mean ankle ROM generated at the crank angle of 20° and 160° between fixed-ankle QH stimulation and free-ankle QHT stimulation (p < 0.001 and p = 0.001, respectively), free-ankle QH stimulation and free-ankle QHT stimulation (p = 0.018 and p = 0.021, respectively), and fixed- and free-ankle QHT stimulation (p = 0.002 and p = 0.025, respectively) (Fig. 4c). There were also significant differences in the mean ankle ROM at the crank angle of 140° and 180° generated between fixed-ankle QH stimulation and free-ankle QHT stimulation (p = 0.033 and p = 0.004, respectively). At the crank angle of 320°, there were significant differences in the mean ankle ROM generated between fixed-ankle QHT stimulation and fixed-ankle QH stimulation (p = 0.014), and fixed- and free-ankle QH stimulation (p = 0.048). Meanwhile, at the crank angle of 360°, there were significant differences in the mean ankle ROM generated between fixed- and free-ankle QH stimulation (p = 0.015), fixed-ankle QH stimulation, and free-ankle QHT stimulation (p < 0.001), free-ankle QH stimulation and fixed-ankle QHT stimulation (p = 0.007), and fixed- and free-ankle QHT stimulation (p < 0.001).

The present study also revealed that there were significant differences in the mean knee ROM generated between free-ankle QH stimulation and fixed-ankle QHT stimulation (p = 0.016), and fixed- and free-ankle QHT stimulation (p = 0.05) at the crank angle of 340° (Fig. 4b).

## Discussion

### The effect of releasing the ankle joint on the pedal power production

The present study sought to investigate the possible differences in mean and peak pedal POs, and hip, knee, and ankle joint ROMs during FES-evoked cycling with fixed- and free-ankle setup in persons with SCI. The mean pedal POs generated from 0° to 360° crank angle during fixed- and free-ankle FES-evoked cycling in the current study were 1.2–27.1 W and 0.6–28.4 W, respectively. To our knowledge, no studies have as yet investigated the effect of fixed- and free-ankle on the pedal PO during FES-evoked cycling in persons with SCI. However, Hamdan et al. reported that the mean and peak pedal POs achieved by healthy individuals during voluntary recumbent cycling with AFO-constrained movement were 17.2 ± 9.0 W (range 2–36 W) and 27.2 ± 12.0 W (range 6–60 W), respectively^24^. Duffell et al. reported that the magnitude of mechanical power produced by persons with SCI during FES-evoked cycling was 8–35 W^40^. The mean pedal PO revealed in the current study was similar to the previous studies. This finding suggested that free-ankle FES-evoked cycling did not significantly elevate the maximum mechanical PO in persons with SCI from the established PO in the previous studies. This might be due to the muscles of each individual with SCI having their maximum power production capacity. One-to-one comparison may not provide an accurate conclusion; thus, a comparison was made on their normalized power production.

There was a statistically significant interaction between the effect of fixed- and free-ankle, and QH and QHT stimulations during FES-evoked cycling on the mean and peak normalized pedal POs across each slice of 20° crank angle intervals, particularly between free-ankle, and QH and QHT stimulations. These findings suggested that both ankle setup and stimulation modes influence power production during FES-evoked cycling in individuals with SCI. There were also significant differences in the mean and peak normalized pedal POs between free-ankle QH stimulation, fixed-ankle QH stimulation, fixed-ankle QHT stimulation, and free-ankle QHT stimulation across each slice of 20° crank angle intervals. This was not the case in healthy individuals, as reported by a previous study where different ankle constraint movements do not influence altering power production during voluntary recumbent cycling in healthy individuals^24^. This finding suggested that fixed- and free-ankle setups only affected the pedal PO produced during FES-evoked cycling. Unlike persons with SCI, the leg muscles of healthy individuals have the ability to adapt to different ankle positioning during voluntary cycling. Overall, free-ankle QH stimulation produced the lowest mean and peak normalized pedal POs. Free-ankle QHT stimulation produced the highest mean normalized pedal PO, while fixed-ankle QHT stimulation produced the highest peak normalized pedal PO. A significant interaction between free-ankle, and QH and QHT stimulations suggests that releasing the ankle joint without the stimulation of tibialis anterior and triceps surae limits the power transmission to the pedal during FES-evoked cycling in persons with SCI. Mean and peak pedal POs generated by free-ankle QH stimulation were shifted down across each slice of 20° crank angle intervals compared to the other settings. There was a significant loss of mean and peak power at the crank angle of 80° and 140°, respectively during free-ankle QH stimulation. The transmission of power produced by the hamstring muscles to the pedal to overcome the dead pedal position (0° /360° and 180° crank angle)^8^ lost at the ankle joint during free-ankle QH stimulation. Fixed-ankle QH stimulation was shown to produce higher pedal PO than free-ankle QH stimulation. This is because solid AFO maintained the legs in the sagittal plane^15^ to optimize the power transmission^41^ to the pedal^8^ during FES-evoked cycling in these individuals. The addition of stimulating tibialis anterior and triceps surae during free-ankle FES-evoked cycling, with pedal spindle attached to the top middle part of the foot was shown to significantly elevate the mean normalized pedal PO by 10.3% more than without the stimulation of shank muscles during free-ankle FES-evoked cycling. The addition of stimulating tibialis anterior and triceps surae during fixed-ankle FES-evoked cycling was shown to significantly elevate the peak normalized pedal PO by 14.5% more than without the stimulation of shank muscles during free-ankle FES-evoked cycling. The present study also revealed that the addition of stimulating tibialis anterior and triceps surae during free-ankle FES-evoked cycling was shown to only elevate the mean normalized pedal PO by 0.8% more than fixed-ankle. This finding did not support the theory developed using simulation models whereby the power could be improved by 14% by releasing the ankle joint and stimulating the triceps surae and tibialis anterior, with the tuning of the contact point between the foot and pedal to the relative strength of the ankle plantar flexors of the triceps surae compared to the fixed-ankle joint^32^. However, it was expected that releasing the ankle joint would not lead to a large increase in PO upheld in reality^32^. The pedal POs generated by fixed-ankle QH stimulation and fixed-ankle QHT stimulation showed no significant differences. This finding suggested that the stimulation of the tibialis anterior and triceps surae contributed to no significant increment of pedal PO during fixed-ankle FES-evoked cycling. The present study reported a similar finding to the previous studies, where gastrocnemius produced no significant difference in the mechanical work^29–31^. A non-statistically significant interaction between fixed-ankle, and QH and QHT stimulations found in this study proves that tibialis anterior and triceps surae are a small muscle group that might produce lower power than quadriceps. This further justifies that the primary power source of FES-evoked cycling was the knee extensors of the quadriceps, followed by the knee flexors of the hamstring^13^.

### The effect of releasing ankle joint on the ankle, knee, and hip ROMs

There was no statistically significant interaction between the effect of fixed- and free-ankle, and QH and QHT stimulations during FES-evoked cycling on the ankle, knee, and hip ROMs across each slice of 20° crank angle intervals. These findings suggested that both ankle setup and stimulation modes do not influence altering ankle, knee, and hip ROMs during FES-evoked cycling in individuals with SCI. The present study also suggested that free-ankle FES-evoked cycling resulted in no knee hyperextension. There were also no significant differences in the ankle, knee, and hip joints ROMs during FES-evoked cycling between fixed-ankle QH stimulation, free-ankle QH stimulation, fixed-ankle QHT stimulation, and free-ankle QHT stimulation across each slice of 20° crank angle intervals. However, further analysis showed that there were significant differences in the ankle joint ROM across each slice of 20° crank angle intervals, particularly at the crank angle of 20°, 140°, 160°, 180°, 320°, and 360°. During free-ankle FES-evoked cycling, a significant ankle dorsi- and plantarflexion movement was generated between 140° to 180° and 0°/360° to 20° crank angle, respectively compared to fixed-ankle FES-evoked cycling. These findings suggested that free-ankle FES-evoked cycling produced greater ankle joint ROM than fixed-ankle joint ROM across each slice of 20° crank angle intervals, with the pedal spindle attached to the top middle part of the foot. The combination of shank muscle stimulation and freeing the ankle joint movement was reported to potentially improve ankle flexibility^33^, for therapeutic benefits and hopefully provide a competitive FES-evoked cycling advantage through the PO increment^42^. Even though the use of AFO in the present study has been proved to limit the ankle dorsi- and plantarflexion movement during fixed-ankle FES-evoked cycling, it was however useful to provide shank stability to individuals with SCI that restricts leg movements in the sagittal plane^27^. Unfortunately, the present study did not analyze the ankle, knee, and hip ROMs in the sagittal plane. Future studies could investigate the effects of free- and fixed-ankle FES-evoked cycling on the ankle, knee, and hip ROMs in the sagittal plane.

### Recommendations and limitations

The present study’s findings might be useful for rehab practitioners in maximizing the benefits of FES-evoked cycling, thus maximizing the health of persons with SCI. However, the findings should be interpreted with caution due to low subject numbers and small effect sizes. A short duration of power production during fixed- and free-ankle with QH and QHT stimulations cycling in the present study might be insufficient to maximize the cycling benefits when compared to a longer duration of cycling. A long cycling duration which is commonly practiced by rehabilitation practitioners in individuals with SCI was more likely to maximize muscle strength and endurance. The general purpose of the present study was to establish the interaction between different ankle setups and stimulation modes on power production during FES-evoked, without muscle fatigue consideration. Therefore, one minute of cycling in the present study is crucial to justify that the significant changes in power production were solely due to either fixed- and free-ankle, or QH and QHT stimulations, or both, not because of other factors such as muscle fatigue. Muscle fatigue might take place in a longer duration of FES-evoked cycling. Therefore, further studies are recommended to understand the effect of releasing the ankle joint during FES-evoked cycling with the stimulation of lower leg muscles for a longer duration throughout training sessions among higher subject numbers.

## Data Availability

The datasets used and/or analyzed during the current study are available from the corresponding author upon reasonable request.

## References

[1] Popovic-Maneski L (2018). Assessment of spasticity by a pendulum test in SCI patients who exercise FES cycling or receive only conventional therapy. IEEE Trans. Neural Syst. Rehabil. Eng..

[2] Topp R, Ditmyer M, King K, Doherty K, Hornyak J (2002). The effect of bed rest and potential of prehabilitation on patients in the intensive care unit. AACN Clin. Issues.

[3] Mayson TA, Harris SR (2014). Functional electrical stimulation cycling in youth with spinal cord injury: A review of intervention studies. J. Spinal Cord Med..

[4] Kahn NN, Feldman SP, Bauman WA (2010). Lower-extremity functional electrical stimulation decreases platelet aggregation and blood coagulation in persons with chronic spinal cord injury: A pilot study. J. Spinal Cord Med..

[5] Johnston TE, Modlesky CM, Betz RR, Lauer RT (2011). Muscle changes following cycling and/or electrical stimulation in pediatric spinal cord injury. Arch. Phys. Med. Rehabil..

[6] Wiesener C, Schauer T (2017). The Cybathlon RehaBike inertial-sensor-driven functional electrical stimulation cycling by team Hasomed. IEEE Robot. Autom. Mag..

[7] Schauer T (2017). Sensing motion and muscle activity for feedback control of functional electrical stimulation: Ten years of experience in Berlin. Annu. Rev. Control.

[8] Laubacher M, Aksöz EA, Bersch I, Hunt KJ (2017). The road to Cybathlon 2016-Functional electrical stimulation cycling Team IRPT SPZ. Eur. J. Transl. Myol..

[9] Ragnarsson KT (1988). Clinical evaluation of computerized functional electrical stimulation after spinal cord injury: A multicenter pilot study. Arch. Phys. Med. Rehabil..

[10] Hunt KJ, Hosmann D, Grob M, Saengsuwan J (2013). Metabolic efficiency of volitional and electrically stimulated cycling in able-bodied subjects. Med. Eng. Phys..

[11] Aksöz EA (2018). Stochastically modulated inter-pulse intervals to increase the efficiency of functional electrical stimulation cycling. J. Rehabil. Assist. Technol. Eng..

[12] Metani A, Popović-Maneski L, Mateo S, Lemahieu L, Bergeron V (2017). Functional electrical stimulation cycling strategies tested during preparation for the first Cybathlon competition: A practical report from team ENS de Lyon. Eur. J. Transl. Myol..

[13] Szecsi J, Straube A, Fornusek C (2014). A biomechanical cause of low power production during FES cycling of subjects with SCI. J. Neuroeng. Rehabil..

[14] Duffell LD, Donaldson NDN, Newham DJ (2009). Why is the metabolic efficiency of FES cycling low?. IEEE Trans. Neural Syst. Rehabil. Eng..

[15] Tong, R. K. Y. *et al.* How to prepare a person with complete spinal cord injury to use surface electrodes for FES trike cycling. In *2017 International Conference on Rehabilitation Robotics (ICORR)* 801–805. 10.1109/ICORR.2017.8009346 (IEEE, 2017). 10.1109/ICORR.2017.8009346 28813918

[16] Bakkum AJT, de Groot S, van der Woude LHV, Janssen TWJ (2012). The effects of hybrid cycle training in inactive people with long-term spinal cord injury: Design of a multicenter randomized controlled trial. Disabil. Rehabil..

[17] Hunt KJ, Fang J, Saengsuwan J, Grob M, Laubacher M (2012). On the efficiency of FES cycling: A framework and systematic review. Technol. Health Care.

[18] Davis GM, Hamzaid NA, Fornusek C (2008). Cardiorespiratory, metabolic, and biomechanical responses during functional electrical stimulation leg exercise: Health and fitness benefits. Artif. Organs.

[19] Hamdan, P. N. F., Teo, K., Hamzaid, N. A., Usman, J. & Razman, R. The effects of releasing ankle joint on pedal force and power production during electrically stimulated cycling in paraplegic individuals: A pilot study. In *3rd International Conference on Movement, Health and Exercise: Engineering Oympic Success: From Theory to Practice*, Vol. 58, 129–130 (2017).

[20] Martin JC, Brown NAT (2009). Joint-specific power production and fatigue during maximal cycling. J. Biomech..

[21] Gregor SM (2002). Lower extremity general muscle moment patterns in healthy individuals during recumbent cycling. Clin. Biomech..

[22] Gregor RJ, Broker JP, Ryan MM (1991). The biomechanics of cycling. Exerc. Sport Sci. Rev..

[23] Winter DA (1991). Biomechanics and Motor Control of Human Gait: Normal, Elderly and Pathological.

[24] Hamdan PNF (2018). Variations of ankle-foot orthosis-constrained movements increase ankle range of movement while maintaining power output of recumbent cycling. Biomed. Tech..

[25] Watanabe T, Tadano T (2018). Experimental tests of a prototype of IMU-based closed-loop fuzzy control system for mobile FES cycling with pedaling wheelchair. IEICE Trans. Inf. Syst..

[26] Wiesener C, Ruppin S, Schauer T (2016). Robust discrimination of flexion and extension phases for mobile functional electrical stimulation (FES) induced cycling in paraplegics. Int. Fed. Autom. Control.

[27] Trumbower RD, Faghri PD (2005). Kinematic analyses of semireclined leg cycling in able-bodied and spinal cord injured individuals. Spinal Cord.

[28] Ferrante, S. *et al.* Quantitative evaluation of stimulation patterns for FES cycling. In *10th Annual Conference of the International FES Society* 2–4 (2005).

[29] Hakansson NA, Hull ML (2010). The effects of stimulating lower leg muscles on the mechanical work and metabolic response in functional electrically stimulated pedaling. IEEE Trans. Neural Syst. Rehabil. Eng..

[30] Hakansson NA, Hull ML (2009). Muscle stimulation waveform timing patterns for upper and lower leg muscle groups to increase muscular endurance in functional electrical stimulation pedaling using a forward dynamic model. IEEE Trans. Biomed. Eng..

[31] Hakansson NA, Hull ML (2007). Influence of pedaling rate on muscle mechanical energy in low power recumbent pedaling using forward dynamic simulations. IEEE Trans. Neural Syst. Rehabil. Eng..

[32] van Soest AJ, Gföhler M, Richard Casius LJ (2005). Consequences of ankle joint fixation on FES cycling power output: A simulation study. Med. Sci. Sport. Exerc..

[33] Fornusek C, Davis GM, Baek I (2012). Stimulation of shank muscles during functional electrical stimulation cycling increases ankle excursion in individuals with spinal cord injury. Arch. Phys. Med. Rehabil..

[34] Dzulkifli MA, Hamzaid NA, Davis GM, Hasnan N (2018). Neural network-based muscle torque estimation using mechanomyography during electrically-evoked knee extension and standing in spinal cord injury. Front. Neurorobot..

[35] Hamzaid NA, Pithon KR, Smith RM, Davis GM (2012). Functional electrical stimulation elliptical stepping versus cycling in spinal cord-injured individuals. Clin. Biomech..

[36] Kearney RS, Lamb SE, Achten J, Parsons NR, Costa ML (2011). In-shoe plantar pressures within ankle-foot orthoses: Implications for the management of achilles tendon ruptures. Am. J. Sports Med..

[37] Fornusek C, Sinclair PJ, Davis GM (2007). The force-velocity relationship of paralyzed quadriceps muscles during functional electrical stimulation cycling. Neuromodulation.

[38] Matsunaga T, Shimada Y, Sato K (1999). Muscle fatigue from intermittent stimulation with low and high frequency electrical pulses. Arch. Phys. Med. Rehabil..

[39] Szecsi J, Straube A, Fornusek C (2014). Leg general muscle moment and power patterns in able-bodied subjects during recumbent cycle ergometry with ankle immobilization. Med. Eng. Phys..

[40] Duffell LD, de Donaldson NN, Newham DJ (2010). Power output during functional electrically stimulated cycling in trained spinal cord injured people. Neuromodulation.

[41] McDaniel J, Lombardo LM, Foglyano KM, Marasco PD, Triolo RJ (2017). Setting the pace: Insights and advancements gained while preparing for an FES bike race. J. Neuroeng. Rehabil..

[42] Hamdan PNF, Hamzaid NA, Abd Razak NA, Hasnan N (2020). Contributions of the Cybathlon championship to the literature on functional electrical stimulation cycling among individuals with spinal cord injury: A bibliometric review. J. Sport Health Sci..

